# The Concurrent Use of Gabapentin and Opioid Analgesia in Burns Patients

**DOI:** 10.5812/atr.6636

**Published:** 2012-08-21

**Authors:** Fiona McClenaghan

**Affiliations:** 1 Oral and Maxillofacial Department, Royal London Hospital, London, UK

**Keywords:** Burns, Gabapentin, Opioid Analgescics

**Dear Editor,**

I read with interest the study by Rimaz et al. (1) which showed a statistically significant reduction in visual analogue pain scores and morphine consumption in a double blind randomised setting with 25 patients in each wing. This is not the first study to suggest that in tackling the complex mechanism of post-burn hypersensitivity, as yet not fully understood but thought to be similar to post-operative pain hypersensitivity; analgesics with different but synergistic effects may prove superior to opioid analgesia alone (2, 3). Although in common use for neuropathic pain worldwide gabapentin has only relatively recently been taken up by plastic surgeons for use in acute burn pain both pre and post-surgical debridement. Although the study undoubtedly shows promising results in terms of a significant decrease in both pain scores and morphine consumption, there are some difficulties which must be addressed prior to gabapentin potentially becoming an accepted adjunct to opioid analgesia in the treatment of acute burns pain. As is often the case with the introduction of adjuvant drug treatment studies are difficult to compare due to the inherent variability of drug dosage, regimen, and use of adjuvant drugs and length of treatment. A similar study looking at the effects of gabapentin on morphine consumption in burns patients by Cuignet et al. (2), although reaching the same overall conclusions as Rimaz et al. (1) used a very different dosing regimen; a single dose of 1200mg gabapentin pre-operatively (1) and 2400mg daily from burn day 3-24 and results recorded for 21 days pre and post-surgical debridement (2) respectively. Additionally, in a further case series using gabapentin for acute burn pain Gray et al. (3) used regimens as varied as 1000mg four times per day with concurrent oxycodone and 300mg three times per day are described.

Therefore although several studies have now shown gabapentin to be a beneficial addition to an opioid analgesia regimen in burns patients (1-3) what is required is collaborative research effort in order to determine the lowest possible dose of gabapentin to achieve maximal pain relief but with minimal side effects. This will allow a future systematic review with significant power to change recommended practice should future research continue to produce positive results. Although Rimaz et al. (1) found no side effects with the 1200mg single pre-operative dose of gabapentin, it must not be forgotten that gabapentin is a drug which can cause significant side effects even if only used for a short period of time, especially at the relatively high doses recommended by Cuignet et al. (2) Multicentre studies of gabapentin in post-herpetic neuralgia and painful diabetic neuropathy have shown significant side effects reporting day-time somnolence of up to 27% (4) and dizziness at 24% (5) which must be balanced with the potential analgesic benefit of gabapentin in burns patients. In the interests of burns patients world-wide is it hoped that further collaborative research will follow promptly to advance a treatment which, although currently in its infancy, has the potential to alleviate the significant pain that many such patients currently suffer.
